# Supplementary material for: Seroepidemiology of enterovirus A71 infection in prospective cohort studies of children in southern China, 2013-2018
Source: Nat Commun. 2022 Nov 26;13:7280. doi: 10.1038/s41467-022-34992-1 (PMC9701185; doi:10.1038/s41467-022-34992-1)
Supplement: Supplementary file 1 — Supplementary Information [file 41467_2022_34992_MOESM1_ESM.pdf]

Supplementary Appendix

Seroepidemiology of children with enterovirus A71 infections in southern China: longitudinal, population-based cohort studies, 2013-2018

Juan Yang[1,14]; Qiaohong Liao[1,2,14]; Kaiwei Luo[3,14]; Fengfeng Liu[2,14]; Yonghong Zhou[1,14]; Gang Zou[4,14]; Wei Huang[3]; Shuanbao Yu[2]; Xianglin Wei[1], Jiaxin Zhou[1]; Bingbing Dai[5]; Qi Qiu[1]; Ralf Altmeyer[4,6]; Hongan Hu[5]; Juliette Paireau[7,8]; Li Luo[2]; Lidong Gao[3]; Birgit Nikolay[7]; Shixiong Hu[3]; Weijia Xing[2,9]; Peng Wu[10]; H. Rogier van Doorn[11,12]; Peter W. Horby[13]; Peter Simmonds[12]; Gabriel M Leung[10]; Benjamin J. Cowling[10]; Simon Cauchemez[7]; Hongjie Yu[1]*

Author Affiliations:

1.School of Public Health, Fudan University, Key Laboratory of Public Health Safety, Ministry of Education, Shanghai, China

2.Key Laboratory of Surveillance and Early-warning on Infectious Disease, Division of Infectious Disease, Chinese Center for Disease Control and Prevention, Beijing, China

3. Hunan Provincial Center for Disease Control and Prevention (Workstation for Emerging Infectious Disease Control and Prevention, Chinese Academy of Medical Sciences), Changsha, China

4.Institut Pasteur of Shanghai, Chinese Academy of Sciences, Shanghai, China

5.Anhua County Center for Disease Control and Prevention, Yiyang, China

6. Medusa Therapeutics Limited, Hong Kong Special Administrative Region, Hong Kong, China

7.Mathematical Modelling of Infectious Diseases Unit, Institut Pasteur, Université Paris Cité, UMR2000, CNRS, 75015 Paris, France

8.Infectious Diseases Department, Santé publique France, Saint-Maurice, France

9.School of Public Health, Shandong First Medical University & Shandong Academy of Medical Sciences, Tai'an, China

10.WHO Collaborating Centre for Infectious Disease Epidemiology and Control, School of Public Health, Li Ka Shing Faculty of Medicine, The University of Hong Kong, Hong Kong Special Administrative Region, Hong Kong, China

11.Oxford University Clinical Research Unit, Hospital for Tropical Diseases, Ho Chi Minh City, Vietnam

12.Nuffield Department of Medicine, University of Oxford, Oxford, UK

13.Centre for Tropical Medicine and Global Health, Nuffield Department of Medicine, University of Oxford, Oxford, UK

14. Contributed equally to this work.

*Corresponding author to Prof. Hongjie Yu, yhj@fudan.edu.cn

School of Public Health, Fudan University, Key Laboratory of Public Health Safety, Ministry of Education, Shanghai, China

Table of Contents

Supplementary Methods **3**

1. **EV-A71 vaccination** 3

   Table 1. National EV-A71 vaccine supplies and estimated vaccination coverage rate 3

2. **Selection of study sites** 4

   Table 2. Population size at the study sites in 2013 4

3. **Study participants in the Children cohort** 4

   Table 3. Sample size requirement for 1- to 9-year age groups 5

4. **Baseline visit** 5
5. **Follow-up visits** 5
6. **Blood sample collection and storage** 5
7. **Sampling for lab test** 6

   Table 4. Distribution of participants aged 1-9 years and the sampling ratio 7

8. **Laboratory procedures** 9

Supplementary Figures **10**

Figure 1. Time series of EV-A71 virus activity during 2013-2016, surveillance data in Anhua County[4] 10

Figure 2. Seroprevalence for those who participated in the semi-annual visits in the Children cohort. 11

Figure 3. New infection for those who participated in the semi-annual visits in the Children cohort (A newly infected individual will be defined as an individual whose titres increased from below to above the infection cut-offs). 12

Figure 4. The proportion of susceptible populations and new infections of EV-A71 (cut-off titre 16), and incidence of EV-A71–related hand, foot, and mouth diseases stratified by sex. 13

Figure 5. Comparison of probability of returning to be susceptible to EV-A71 in participants who were infected between groups with different initial antibody titres at baseline. 14

Figure 6. EV-A71 geometric mean titres (GMT) and probability of returning to be susceptible to EV-A71. 15

Supplementary Tables **16**

Table 5. Comparison of baseline characteristics of participants in the Children cohort (participants with lab test done vs. those for whom we did not do lab tests) 16

Table 6. Sex-stratified analysis of characteristics of participants with lab test results being performed at recruitment in Children cohort 18

Table 7. Seroprevalence of EV-A71 antibody under a cutoff titre of 1:8, 1:16 and 1:32, separately 20

Table 8. Incidence of EV-A71 infections under a cutoff titre of 1:8, 1:16 and 1:32, separately 21

Table 9. The sample size of each panel in figure 2 and figure 3 in the main text 22

Supplementary Notes **24**

Supplementary References **25**

Supplementary Methods

1. EV-A71 vaccination

We extracted data on the EV-A71 vaccine lot release during 2016-2020 from the National Institutes for Food and Drug Control website (https://www.nifdc.org.cn/nifdc/data/index.html). In addition, we collected the age-specific population size in 2016 and estimated the age structure in the following years. According to package inserts, vaccines produced by the Institute of Medical Biology Chinese Academy of Medical Sciences are eligible for 6- to 71-month-old children, whereas vaccines from Sinovac Biotech or Wuhan Institute of Biological Products are only eligible for 6- to 35-month-old children between 2016 and 2020.

We assumed two scenarios according to the eligible age groups for the current three licenced EV-A71 vaccines. First, all of the vaccines aimed at 6- to 71-month-old children are used in children aged 3-5 years old, and all of the vaccines aimed at 6- to 35-month-old children are used in children aged 0-2 years old. Second, all three types of vaccines are administered to children aged 0-2 years old. Under the first scenario, on average, the estimated coverage rate of the full course (i.e., two doses) is 9.3% for children aged 3-5 years old during 2016-2020 and is 9.5% for children aged 0-2 years old. Under the second scenario, the estimated coverage rate is 17.6% for children aged 0-2 years old.

Table 1. National EV-A71 vaccine supplies and estimated vaccination coverage rate

Year	Institute of Medical Biology Chinese Academy of Medical Sciences (6-71 months)	Size of population aged 3-5 years old, 2016	Maximum estimated vaccination coverage rate for population aged 3-5 years old	Sinovac Biotech/Wuhan Institute of Biological Products (6-35 months)	Size of population aged 0-2 years old, 2016	Estimated coverage rate of vaccination with Sinovac Biotech/Wuhan Institute of Biological Products for population aged 0-2 years old	Maximum estimated vaccination coverage rate for population aged 0-2 years old
2016	4,154,932	44,221,814	4.7%	4,599,647	50,662,947	4.5%	8.6%
2017	7,978,000	44,221,814	9.0%	6,944,845	50,662,947	6.9%	14.7%
2018	11,611,382	44,221,814	13.1%	18,441,061	50,662,947	18.29%	29.7%
2019	10,208,520	44,221,814	11.5%	9,827,127	50,662,947	9.7%	19.8%
2020	6,975,370	44,221,814	7.9%	8,538,137	50,662,947	8.4%	15.3%
Average	/	/	9.3%	/	/	9.5%	17.6%

2. Selection of study sites

In 2013, the total population in Anhua County was 1,027,585, of whom 110,994 (10.8%) were children aged <10 years and 69,336 (6.7%) were aged <5 years. Among 23 townships in Anhua, we selected three townships for study participant enrolment, i.e., Tianzhuang, Jiangnan and Qingtang (map shown elsewhere[1]). The criteria for study site selection included the following:

(1) a sufficient number of children aged 1-9 years to meet the required sample size (Supplementary Table 1 shows the population size for study sites);

(2) low mobility of children out of the townships and between townships to maintain follow-ups;

(3) relatively convenient transportation to ensure participation in "fixed-post" follow-up visits;

(4) local township health centres have a strong capacity to implement rigorous scientific studies, with experiences in conducting national research programs and a strong coordination ability.

Table 2. Population size at the study sites in 2013

Township	Total	0 year	1 year	2 years	3 years	4 years	5 years	6-9 years
Jiangnan	61,018	905	790	784	791	870	621	1921
Qingtang	63,305	837	857	736	795	839	568	1949
Tianzhuang	32,016	495	482	441	354	342	389	1189
Total	156,339	2237	2129	1961	1940	2051	1578	5059

3. Study participants in the Children cohort

(1) Inclusion criteria

Participants had to meet the following inclusion criteria: 1-9 years of age at enrolment (i.e., 12-23 months for the 1-year group, 24-35 months for the 2-year group, 36-47 months for the 3-year group, etc.); reside in the study site for at least 3 months.

(2) Sampling and enrolment procedures

- Sample size

A sample size of 553 participants for each group (including the 1-year, 2-year, 3-year, 4-year, 5-year, and 6-9-year age groups) would permit estimation of the incidence rate of EV-A71 infections as 10% with a 5% statistical significance level and 2.5% marginal error. Based on the pilot study, we assumed dropout rates of 21% for the 1-year and 2-year age groups and 15% for the other age groups. Accordingly, the adjusted sample sizes were 700 separately for the 1-year and 2-year age groups and 650 separately for the other age groups.

- Sampling and enrolment for the 1- to 9-year age group

For the 1-9 years of age group, the sample size for each study township (shown in Supplementary Table 2) was determined using the proportionate stratified sampling method. Within each township, simple random sampling was used for the selection of children aged 1-9 years.

Table 3. Sample size requirement for 1- to 9-year age groups

Township	1 year	2 years	3 years	4 years	5 years	6-9 years	Total
Jiangnan	257	266	229	218	232	235	1437
Qingtang	312	326	317	333	314	309	1911
Tianzhuang	131	108	105	98	104	106	653
Total	700	700	650	650	650	650	4000

Each study site generated a list of registered residents aged 1-9 years in the township who were eligible for enrolment. Considering the willingness to participate observed in the pilot study, 150% of the required number of enrolments will be randomly selected from the list. Potential participants were then approached and invited by well-trained project personnel and/or village doctors in order of random numbers. If selected children were unavailable or declined participation, the next eligible child was approached until sufficient children were successfully recruited in each age group. Enrolment began on 23 September 2013 and was closed on 25 November 2013. We approached 5,996 children and enrolled 4,188 (70%) children in total.

4. Baseline visit

On the same day of enrolment, a baseline serologic survey was completed for all enrolled children, including a venous blood sample (2 ml) drawn from each study participant, and a questionnaire survey on their caregivers.

5. Follow-up visits

- Annual follow-up visits

Two HFMD epidemic seasons per year were observed in Yiyang prefecture, including a major seasonal peak in April-July and a minor seasonal peak in late October-November [2]. Regular follow-up visits were conducted between August and mid-October every year for all enrolled participants aged 1-9 years during 2014-2016, producing paired sera samples to estimate the incidence rates of enterovirus infections for the annual epidemic season.

- Additional semi-annual follow-up visits

To estimate the semi-annual incidence rates of enterovirus infections, an average of 25% of enrolled participants in each age group (n=1047, hereafter called subgroup) were randomly selected to participate in three additional follow-up visits between February and March during 2014-2016.

To summarise, a total of six follow-up visits were conducted for the subgroup, separately between February-March 2014 (hereafter called follow-up visit 1), August-October 2014 (follow-up visit 2), March 2015 (follow-up visit 3), August-October 2015 (follow-up visit 4), March 2016 (follow-up visit 5), and August-November 2016 (follow-up visit 6). Other participants aged 1-9 years only attended follow-up visits 2, 4 and 6.

6. Blood sample collection and storage

At each visit, a venous blood sample (2 ml) was drawn from every study participant, and we obtained umbilical cord blood (2 ml) from neonates at birth. Blood specimens were placed on ice

packs and transported to Anhua CDC for processing. Serum was separated, split into 3 aliquots and temporarily frozen at -70 °C at local CDC laboratories. Serum samples were then transferred on dry ice to Fudan University and stored at -80 °C until testing.

7. Sampling for lab test

Completing the neutralising assays on neutralising antibodies against EV-A71 for all study participants is quite a resource-intensive task. Using multistage proportional stratified random sampling, we selected specimens from 50% of the enrolled participants aged 1-5 years for neutralising assays on neutralising antibodies against EV-A71. Stratification factors included 1) age (1 year, 2 years, 3 years, 4 years, and 5 years); 2) group (subgroup who were required to participate in semi-annual follow-up visits and others who participated in annual follow-up visits); and 3) number of follow-up visits. Supplementary Table 3 shows the distribution of participants aged 1-9 years.

Considering the relatively small study population size in the 6- to 9-year-old age group, lab tests were conducted for all specimens.

In total, the specimens of 2475 participants were used for the lab test.

Table 4. Distribution of participants aged 1-9 years and the sampling ratio

	No. of follow-up visits	No. participants and sampling for lab-test on EV-A71 antibody	Age group						Total
			1 year	2 year	3 year	4 year	5 year	6-9 year	
Those who participated to annual follow-up visits	1	No. all participants	40	49	28	29	25	26	197
		No. participants with lab test performed	20	24	14	15	13	26	112
		Sampling ratio*	50%	49%	50%	52%	52%	100%	57%
	2	No. all participants	61	47	34	43	36	44	265
		No. participants with lab test performed	31	27	18	22	18	44	160
		Sampling ratio*	50%	57%	53%	51%	50%	100%	60%
	3	No. all participants	87	100	64	82	84	115	532
		No. participants with lab test performed	44	49	33	41	42	115	324
		Sampling ratio*	51%	49%	52%	50%	50%	100%	61%
	4	No. all participants	344	368	357	339	343	396	2147
		No. participants with lab test performed	174	182	180	169	169	396	1270
		Sampling ratio*	50%	49%	50%	50%	49%	100%	59%
Subgroup who participated to semi-annual follow-up visits	2	No. all participants	7	3	2	2	5	5	24
		No. participants with lab test performed	3	2	2	1	3	5	16
		Sampling ratio*	43%	67%	100%	50%	60%	100%	67%
	3	No. all participants	10	5	5	7	2	2	31
		No. participants with lab test performed	5	2	3	4	1	2	17
		Sampling ratio*	50%	40%	60%	57%	50%	100%	55%
	4	No. all participants	11	9	4	5	5	3	37
		No. participants with lab test performed	5	4	2	3	3	3	20
		Sampling ratio*	45%	44%	50%	60%	60%	100%	54%
	5	No. all participants	10	16	13	11	11	10	71

		No. participants with lab test performed	5	9	7	6	5	10	42
		Sampling ratio*	50%	56%	54%	55%	45%	100%	59%
		No. all participants	24	29	31	17	17	19	137
	6	No. participants with lab test performed	12	14	16	9	8	19	78
		Sampling ratio*	50%	48%	52%	53%	47%	100%	57%
		No. all participants	115	115	114	129	137	137	747
	7	No. participants with lab test performed	56	55	57	63	68	137	436
		Sampling ratio*	49%	48%	50%	49%	50%	100%	58%
		No. all participants	709	741	652	664	665	757	4188
Total		No. participants with lab test performed	355	368	332	333	330	757	2475
		Sampling ratio*	50%	50%	51%	50%	50%	100%	59%

*Sampling ratio=no. of those with lab test performed/no. of all participants in each stratification.

8. Laboratory procedures

(1) Virus strains

The EV-A71 strain (FY573, GenBank accession number: HM064456.1) used in this study was isolated from a child with HFMD from Fuyang city of Anhui province in 2008. The EV-A71 isolates from Anhua County and FY573 clustered within their respective subgenogroups C4, which were the main circulating strains of mainland China in recent years.

(2) Neutralising assays

Neutralising antibody titres against EV-A71 were detected with a neutralisation test on human rhabdomyosarcoma (RD) cell lines. Serum samples were inactivated at 56 °C for 30 min and then serially diluted 4-fold from 1:8 to 1:2048 with duplicate wells of each dilution. Fifty microlitres of serum dilution was mixed with an equal volume (50 μl) of the virus solution (100 TCID50/50 μl) and incubated at 37 °C for 2 hours in the presence of 5% CO_2. Then, 100 μl of RD cell suspension (2×10^5 cells/ml) was seeded into each well of a 96-well plate and incubated at 37 °C for 7 days in 5% CO_2. Serum toxicity was determined by incubation of the lowest serum dilution with cells without virus. A cell control, positive antibody control, virus control and serum toxicity control (only contain serum and cells) were set up on each plate. A virus back titration and EV-A71 NAb standards from National Institutes for Food and Drug Control (strongly positive, weakly positive and negative) were used for quality control. Neutralising titres were defined as the reciprocal of the highest dilution capable of inhibiting 50% of the CPE and calculated using the Karber method[3]. Neutralisation titres <8 or >2048 were assigned a value of 4 or 4096, respectively.

Supplementary Figures

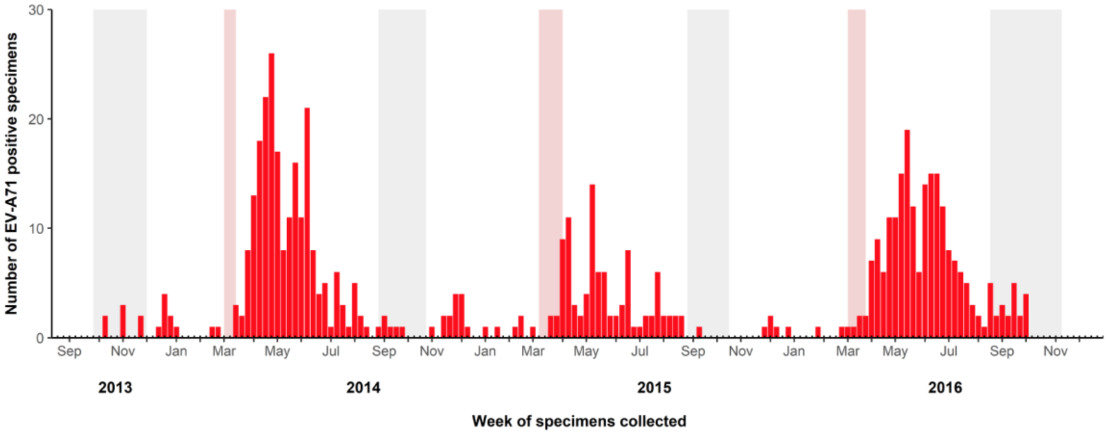

Figure 1. Time series of EV-A71 virus activity during 2013-2016, surveillance data in Anhua County[4].

The red bar denotes the weekly percentage of EV-A71-positive patients, the grey shadow denotes the annual follow-up periods, and the pink shadow denotes the semi-annual follow-up periods.

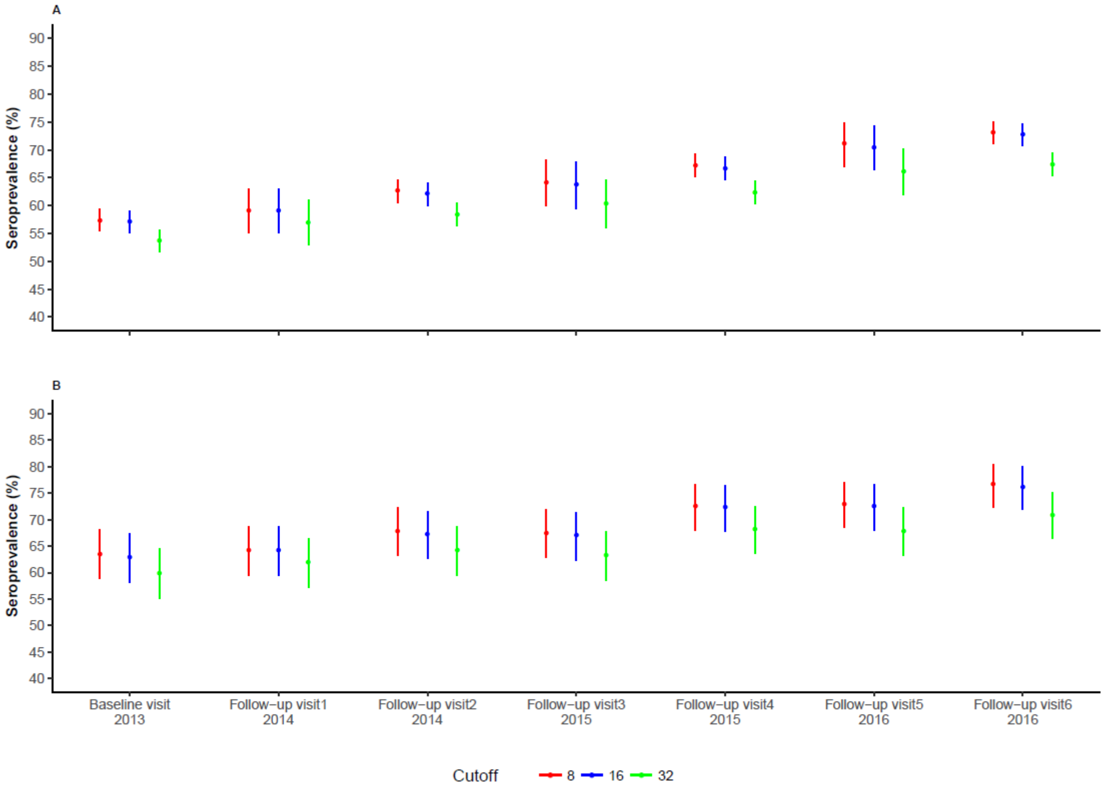

Figure 2. Seroprevalence for those who participated in the semi-annual visits in the Children cohort.

Panel A: Seroprevalence for all study participants who participated in the semi-annual visits (n=609, points represent mean seroprevalence, error bars represent corresponding 95% CI). Panel B: Seroprevalence for those who participated in all the semi-annual visits (i.e., full follow-up group) (n=436, points represent mean seroprevalence, error bars represent corresponding 95% CI).

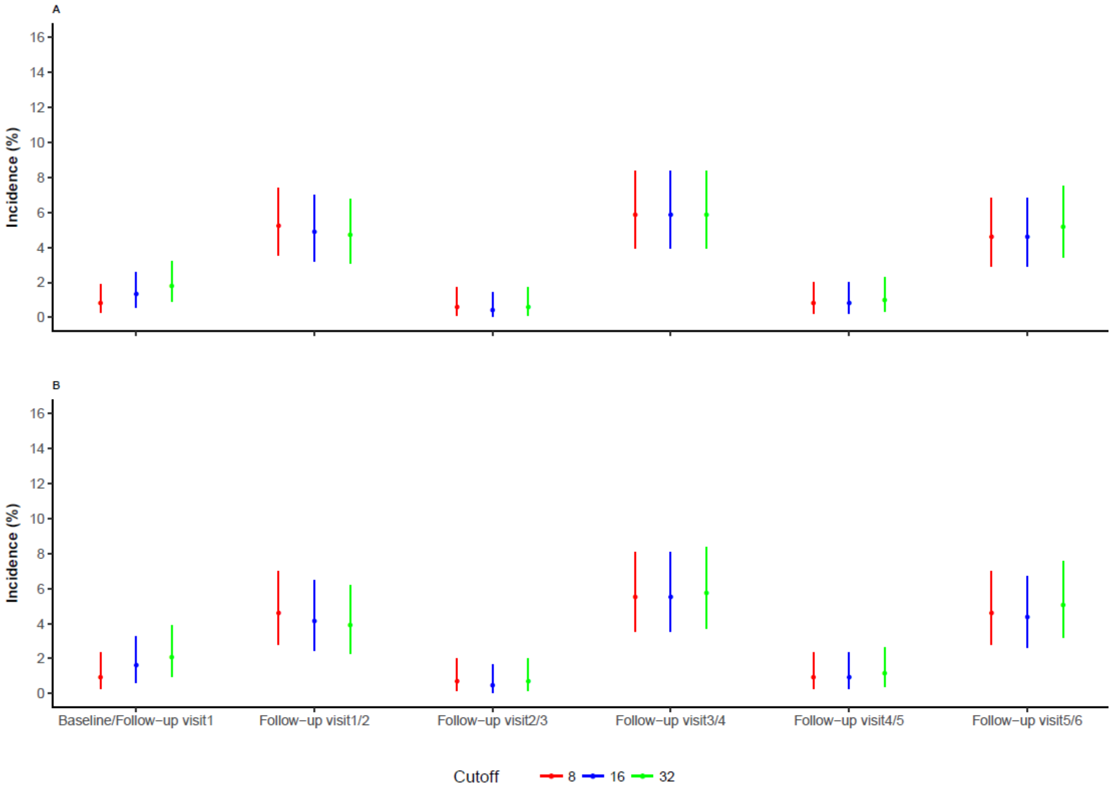

Figure 3. New infection for those who participated in the semi-annual visits in the Children cohort (A newly infected individual will be defined as an individual whose titres increased from below to above the infection cut-offs).

The incidence of EV-A71 infections by serology was calculated for each contiguous visit (e.g., baseline and follow-up visit 1, follow-up visit 1 and follow-up visit 2). Panel A: incidence for all study participants who participated in the semi-annual visits (n=609, points represent mean seroincidence, error bars represent corresponding 95% CI). Panel B: incidence for those who participated in all the semi-annual visits (i.e., full follow-up group) (n=436, points represent mean seroincidence, error bars represent corresponding 95% CI).

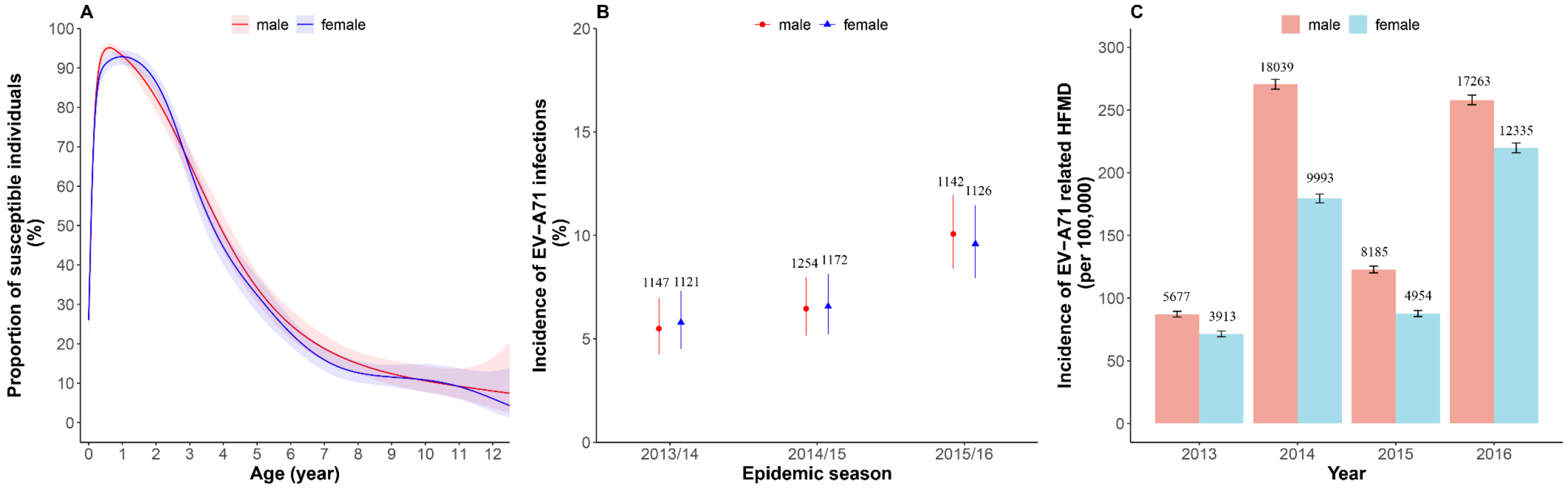

Figure 4. The proportion of susceptible populations and new infections of EV-A71 (cut-off titre 16), and incidence of EV-A71–related hand, foot, and mouth diseases stratified by sex.

Panel A: proportion of susceptible populations for all study participants in Children and Neonate cohort (line represents predicted mean proportion, whereas shadow represents 95% CI). Panel B: incidence of EV-A71 infections for those study participants with paired sera before and after HFMD epidemics (points represent observed seroincidence, error bar represents corresponding 95% CI). The numerical values above the upper limit of 95% CI represent the number of paired sera. Panel C: Incidence of EV-A71-associated hand, foot, and mouth diseases in Hunan province (points represent observed incidence, error bar represents corresponding 95% CI). The numerical values above the bar represent the number of EV-A71 – related HFMD.

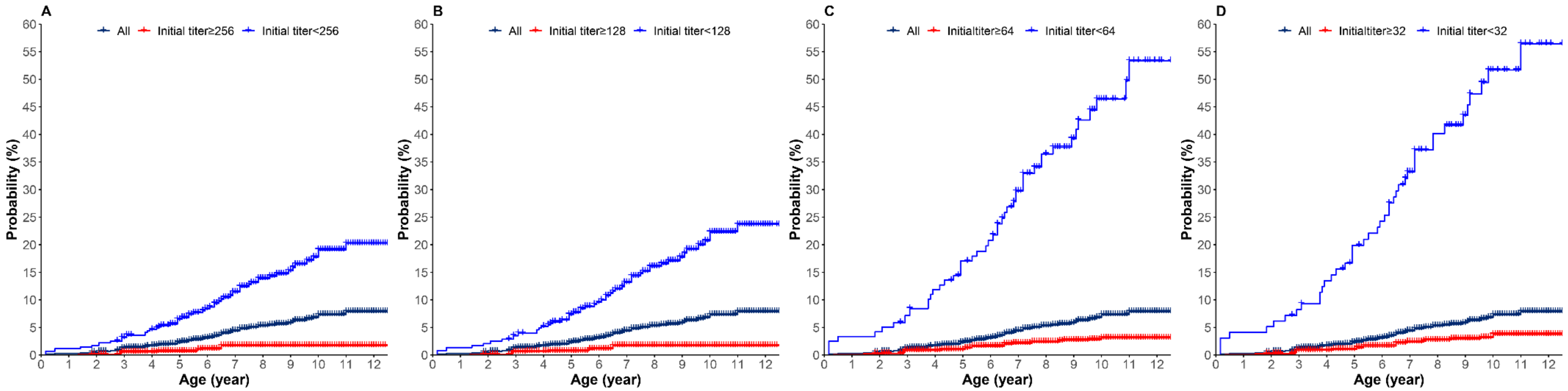

Figure 5. Comparison of probability of returning to be susceptible to EV-A71 in participants who were infected between groups with different initial antibody titres at baseline.

Panel A: a cutoff titre of 256; Panel B: a cutoff titre of 128; Panel C: a cutoff titre of 64; Panel D: a cutoff titre of 32.

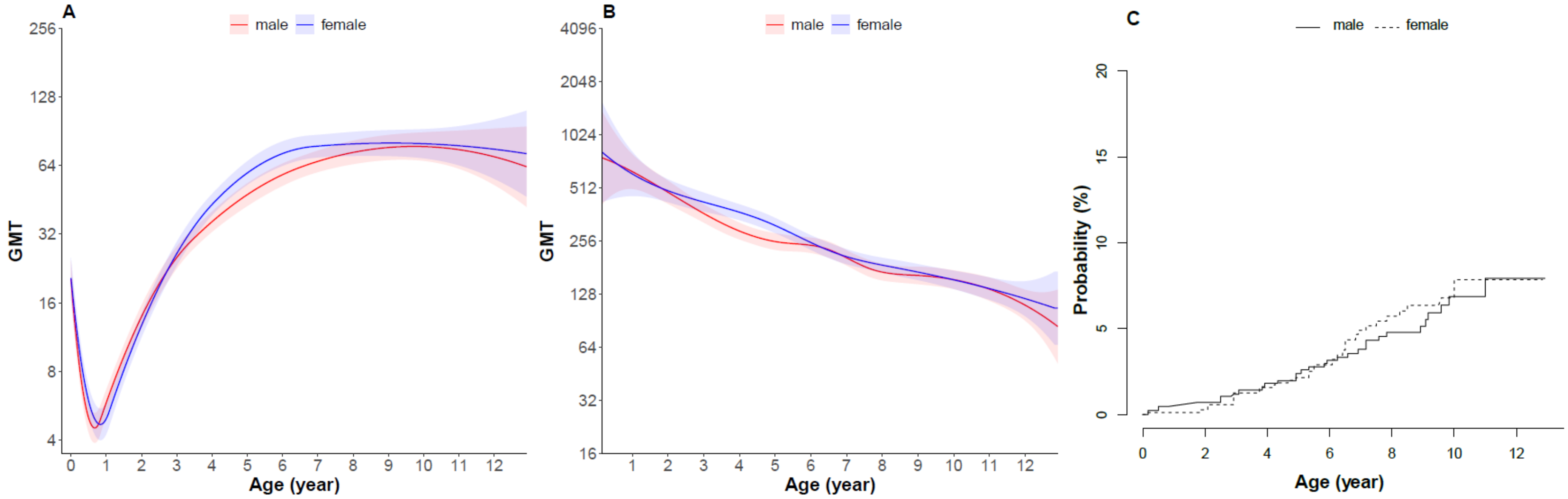

Figure 6. EV-A71 geometric mean titres (GMT) and probability of returning to be susceptible to EV-A71.

Panel A: Age- and sex-specific GMT for all study participants in Children and Neonates cohort (line represents predicted GMT, whereas shadow represents 95% CI). Panel B: Age- and sex-specific GMT in the seropositive participants, excluding maternal antibody titres (line represents predicted GMT, whereas shadow represents 95% CI). Panel C: Kaplan–Meier plot of the probability of loss of immunity in participants who were infected.

Supplementary Tables

Table 5. Comparison of baseline characteristics of participants in the Children cohort (participants with lab test done vs. those for whom we did not do lab tests)

Characteristics	All participants (N=4188)		
	Lab test completed (n=2475)	Lab test incomplete (n=1713)	*P* value
Male, sex	1246/2475 (50%)	869/1713 (51%)	0.83
Age at baseline (year)			
1	355/2475 (14%)	354/1713 (21%)	
2	368/2475 (15%)	373/1713 (22%)	
3	332/2475 (13%)	320/1713 (19%)	
4	333/2475 (13%)	331/1713 (19%)	
5	330/2475 (13%)	335/1713 (20%)	< 0.001
6	283/2475 (11%)	0/1713 (0%)	
7	205/2475 (8%)	0/1713 (0%)	
8	162/2475 (7%)	0/1713 (0%)	
9	107/2475 (4%)	0/1713 (0%)	
Ethnicity (Han)	2474/2475 (100%)	1712/1713 (100%)	1.00
Birth			
Full-term birth	2344/2474 (95%)	1616/1713 (94%)	
Preterm birth	105/2474 (4%)	89/1713 (5%)	0.055
Post-term birth	25/2474 (1%)	8/1713 (0%)	
Delivery mode			
Natural childbirth	1577/2474 (64%)	1077/1713 (63%)	0.59
Caesarean	897/2474 (36%)	636/1713 (37%)	
Birth weight (kilograms, mean, SD)	3.2 (0.5)	3.3 (0.5)	0.442*
Underlying diseases	16/2474 (1%)	11/1712 (1%)	1.00
Number of household members			
2	64/2475 (3%)	40/1713 (2%)	
3	431/2475 (17%)	267/1713 (16%)	
4	695/2475 (28%)	460/1713 (27%)	0.23
5	531/2475 (21%)	374/1713 (22%)	
≥6	754/2475 (30%)	572/1713 (33 %)	
Persons who care for study participants at home (≥60% time)			
Parents	1325/2475 (54%)	946/1713 (55%)	0.28
Others	1150/2475 (46%)	767/1713 (45%)	
Annual family income (RMB, Yuan)			
<20 000	611/2475 (25%)	382/1713 (22%)	0.11
[20 000, 50 000)	1382/2475 (56%)	963/1713 (56%)	

≥50 000	482/2475 (19%)	368/1713 (21%)

Note: All statistical tests were two-sided. "*" indicates that *p* values were estimated by t test, all other *p* values were estimated by chi-square test.

Table 6. Sex-stratified analysis of characteristics of participants with lab test results being performed at recruitment in Children cohort

**Characteristics**	Total (n=2475)	Male (n=1246)	Female (n= 1229)	*P* value
Age at baseline (year) (n, %)				
1	355 (14)	186 (15)	169 (14)	0.295
2	368 (15)	189 (15)	179 (15)	
3	332 (13)	181 (15)	151 (12)	
4	333 (13)	155 (12)	178 (14)	
5	330 (13)	158 (13)	172 (14)	
6	283 (11)	138 (11)	145 (12)	
7	205 (8)	110 (9)	95 (8)	
8	162 (7)	72 (6)	90 (7)	
9	107 (4)	57 (5)	50 (4)	
Ethnicity (Han) (n, %)	2474 (100)	1246 (100)	1228 (100)	0.995
Township (n, %)				
Tianzhuang (1.5 persons per 10^4 m^2)	586 (24)	288 (23)	298 (24)	0.569
Jiangnan (2.1 persons per 10^4 m^2)	787 (32)	408 (33)	379 (31)	
Qingtang (2.7 persons per 10^4 m^2)	1102 (45)	550 (44)	552 (45)	
Birth mode (n, %)				
Full-term birth	2344 (95)	1186 (95)	1158 (94)	
Preterm birth	105 (4)	50 (4)	55 (4)	0.486
Post-term birth	25 (1)	10 (1)	15 (1)	
Delivery mode (n, %)				
Natural childbirth	1577 (64)	794 (64)	783 (64)	1.000
Caesarean	897 (36)	452 (36)	445 (36)	
Birth weight (kilograms, mean, SD)	3.2 (0.5)	3.3 (0.5)	3.2 (0.5)	<0.001*
Underlying diseases (n, %)	16 (1)	8 (1)	8 (1)	1.000
Number of household members (n, %)				
2	64 (3)	37 (3)	27 (2)	0.139
3	431 (17)	232 (19)	199 (16)	
4	695 (28)	335 (27)	360 (29)	
5	531 (21)	278 (22)	253 (21)	
≥6	754 (30)	364 (29)	390 (32)	
Persons who care for study participants at home (≥60% time) (n, %)				
Parents	1325 (54)	653 (52)	672 (55)	0.275
Others	1150 (46)	593 (48)	557 (45)	
Annual family income (RMB, Yuan) (n, %)				
<20 000	611 (25)	321 (26)	290 (24)	0.227

[20 000, 50 000)	1382 (56)	697 (56)	685 (56)
≥50 000	482 (19)	228 (18)	254 (21)

Note: All statistical tests were two-sided. "*" indicates that *p* values were estimated by t test, all other *p* values were estimated by chi-square test.

Table 7. Seroprevalence of EV-A71 antibody under a cutoff titre of 1:8, 1:16 and 1:32, separately

	**2013**	**2014**	**2015**	**2016**
No. cases	2475	2135	2077	2032
Seroprevalence (cutoff 1:8)				
No. infected	1420	1337	1397	1486
Prevalence (mean, 95%CI) (%)	57 (55, 59)	63 (61, 65)	67 (65, 69)	73 (71, 75)
Seroprevalence (cutoff 1:16)				
No. infected	1412	1326	1386	1478
Prevalence (mean, 95%CI) (%)	57 (55, 59)	62 (60, 64)	67 (65, 69)	73 (71, 75)
Seroprevalence (cutoff 1:32)				
No. infected	1329	1247	1296	1370
Prevalence (mean, 95%CI) (%)	54 (52, 56)	58 (56, 61)	62 (60, 64)	67 (65, 69)
Comparison amongst above three cutoffs of EV-A71 infections				
χ^2 value	8.328	9.495	13.077	20.104
P value	0. 016ǂ	0.009ǂ	0.001ǂ	< 0.001[ǂ]
Comparison amongst two cutoffs of EV-A71 infections (1:8 vs. 1:16)				
χ^2 value	0.040	0.100	0.109	0.061
P value[§]	0.841	0.752	0.741	0.805
Comparison amongst two cutoffs of EV-A71 infections (1:8 vs. 1:32)				
χ^2 value	6.627	7.764	10.558	15.578
P value[§]	0.010[ǂ]	0.005[ǂ]	0.001[ǂ]	< 0.001[ǂ]
Comparison amongst two cutoffs of EV-A71 infections (1:16 vs. 1:32)				
χ^2 value	5.497	5.950	8,335	13.435
p value[§]	0.019	0.015[ǂ]	0.004[ǂ]	< 0.001[ǂ]

Note: All statistical tests were two-sided and *p* values were estimated by chi-square test, and multiple $\chi 2$ comparisons were done with a Bonferroni-adjusted α value if relevant. [§]After Bonferroni adjustment, the significance *p* value was adjusted from 0.05 to 0.017, which means *p* value <0.017 represents significant difference. [ǂ] significant difference.

Table 8. Incidence of EV-A71 infections under a cutoff titre of 1:8, 1:16 and 1:32, separately

	**2013/2014**	**2014/2015**	**2015/2016**
No. cases	2136	1918	1862
Seroincidence (cutoff 1:8)			
No. seroconversion	118	103	145
Incidence (mean, 95%CI) (%)	5.5 (4.6-6.6)	5.4 (4.4-6.5)	7.8 (6.6-9.1)
Seroincidence (cutoff 1:16)			
No. seroconversion	116	104	146
Incidence (mean, 95%CI) (%)	5.4 (4.5-6.5)	5.4 (4.5-6.5)	7.8 (6.7-9.2)
Seroincidence (cutoff 1:32)			
No. seroconversion	124	103	143
Incidence (mean, 95%CI) (%)	5.8 (4.9-6.9)	5.4 (4.4-6.5)	7.7 (6.5-9.0)
Comparison amongst above three cutoffs of incidence of EV-A71 infections			
χ^2 value	0.308	0.007	0.035
P value	0.857	0.997	0.983

Note: All statistical tests were two-sided and *p* values were estimated by chi-square test, and multiple χ2 comparisons were done with a Bonferroni-adjusted α value if relevant. §After Bonferroni adjustment, the significance p value was adjusted from 0.05 to 0.017, which means *p* value <0.017 represents significant difference.

Table 9. The sample size of each panel in figure 2 and figure 3 in the main text

Figure.2a

Survey	Age (year), No. of seronegative participants/No. of total participants												
time	0	1	2	3	4	5	6	7	8	9	10	11	12
2013	77/174	269/355	240/368	174/332	121/333	106/330	69/283	39/205	24/162	21/107	-	-	-
2014	1249/2010	-	201/305	174/305	130/293	89/292	90/319	50/218	36/180	17/140	15/75	-	-
2015	883/1180	340/376	-	172/304	134/289	111/281	65/313	87/313	51/207	32/187	19/136	10/67	-
2016	127/133	261/299	245/344	-	137/302	99/275	92/284	58/288	64/291	43/202	25/181	18/133	11/67
2017	-	21/26	153/233	233/362	-	-	-	-	-	-	-	-	-
2018	-	-	49/66	70/104	-	-	-	-	-	-	-	-	-

Figure.2b

Epidemic	Age, No. of EV-A71 infections identified by serology/No. of total participants														
season	0-2 m	3-4 m	5-6 m	7-11 m	1 y	2 y	3 y	4 y	5 y	6 y	7 y	8 y	9 y	10 y	11 y
2013/14	12/136	1/5	-	-	24/279	25/310	20/292	16/285	19/296	6/257	1/173	1/144	3/91	-	-
2014/15	14/236	7/70	27/116	2/53	4/45	24/265	30/273	19/264	14/259	8/289	1/198	5/166	2/128	1/64	-
2015/16	6/38	1/13	9/34	8/55	40/189	18/90	42/273	29/251	25/258	15/260	16/277	4/185	5/170	3/120	2/55

Figure.2c

Survey	Age, No. of EV-A71-associated HFMD patients/No. of total participants						
time	0-2 m	3-4 m	5-6 m	7-11 m	1-2 y	3-5 y	6-14 y
2013	1/208505	54/139003	183/139003	898/347508	5714/1809128	2337/2400432	288/6946451
2014	63/218738	9/145825	269/145825	1239/364563	15802/1922018	9557/2430062	802/7007680
2015	0/221247	36/147498	140/147498	895/368745	6811/1931711	4567/2465123	581/7035286
2016	31/229500	72/153000	200/153000	2276/382499	14782/1923820	11294/2455418	879/7006022

Figure.3a

Survey time	Age (year), No. of participants												
	0	1	2	3	4	5	6	7	8	9	10	11	12
2013	174	355	368	332	333	330	283	205	162	107	-	-	-
2014	2010	-	305	305	293	292	319	218	180	140	75	-	-
2015	1180	376	-	304	289	281	281	313	207	187	136	67	-
2016	133	299	344	-	302	275	284	288	291	202	181	133	67
2017	-	26	233	362	-	-	-	-	-	-	-	-	-
2018	-	-	66	104	-	-	-	-	-	-	-	-	-

Figure.3b

Survey time	Age (year), No. of participants												
	0	1	2	3	4	5	6	7	8	9	10	11	12
2013	-	83	118	151	200	211	210	159	134	79	-	-	-
2014	23	-	102	133	165	208	230	172	146	125	60	-	-
2015	10	34	-	128	152	169	219	234	161	156	119	57	-
2016	3	30	70	-	121	145	172	215	217	158	152	115	56
2017	-	3	60	70	-	-	-	-	-	-	-	-	-
2018	-	-	16	27	-	-	-	-	-	-	-	-	-

Supplementary Notes

We applied generalized linear mixed models (PROC glimmix in SAS) using B-splines to fit the dynamics of the proportion of susceptible individuals. The model selection including B-spline's degree and knots, and model parameters were based on Akaike Information Criterion (AIC). The original model with a spline of degree two had minimum AIC. However, the observed proportions of susceptible individuals in older age groups were not be captured well by the model. Moreover, the degree of splines is commonly set as three since this degree can depict smooth and natural curves [5]. Therefore, we built a spline of degree three to characterize the gradual change of the susceptible proportion. The residual deviations between observed proportions of susceptible individuals and fitted curve in older age groups were caused by below factors: 1) sample sizes were smaller at older age groups, so the variations were greater than those in younger groups. For example, there were only 67 observations at 12 years of age. 2) We used age-specific (in months) proportions of susceptible individuals to fit the model, but grouped the observed proportions by age of years for clarity.

Supplementary References

1. Gao L, et al. Spectrum of enterovirus serotypes causing uncomplicated hand, foot, and mouth disease and enteroviral diagnostic yield of different clinical samples. Clin Infect Dis 67, 1729-1735 (2018).

2. Luo KW, et al. Hand, Foot, and Mouth Disease in Hunan Province, China, 2009-2014: Epidemiology and Death Risk Factors. PloS one 11, e0167269 (2016).

3. Yang L, et al. A neonatal mouse model for the evaluation of antibodies and vaccines against coxsackievirus A6. Antiviral research 134, 50-57 (2016).

4. Wei XL, et al. The transfer and decay of maternal antibodies against enterovirus A71, and dynamics of antibodies due to later natural infections in Chinese infants: a longitudinal, paired mother-neonate cohort study. Lancet Infectious Diseases 21, 418-426 (2021).

5. Perperoglou A, Sauerbrei W, Abrahamowicz M, Schmid M. A review of spline function procedures in R. BMC medical research methodology 19, 46 (2019).
