# Supplementary material for: Seroepidemiology of enterovirus A71 infection in prospective cohort studies of children in southern China, 2013-2018
Source: Nat Commun. 2022 Nov 26;13:7280. doi: 10.1038/s41467-022-34992-1 (PMC9701185; doi:10.1038/s41467-022-34992-1)
Supplement: Supplementary file 3 — Reporting Summary [file 41467_2022_34992_MOESM3_ESM.pdf]

nature portfolio

Corresponding author(s): Hongjie Yu

Last updated by author(s): Nov 3, 2022

Reporting Summary

Nature Portfolio wishes to improve the reproducibility of the work that we publish. This form provides structure for consistency and transparency in reporting. For further information on Nature Portfolio policies, see our Editorial Policies and the Editorial Policy Checklist.

Statistics

For all statistical analyses, confirm that the following items are present in the figure legend, table legend, main text, or Methods section.

n/a	Confirmed	
☐	☒	The exact sample size (n) for each experimental group/condition, given as a discrete number and unit of measurement
☐	☒	A statement on whether measurements were taken from distinct samples or whether the same sample was measured repeatedly
☐	☒	The statistical test(s) used AND whether they are one- or two-sided<br>*Only common tests should be described solely by name; describe more complex techniques in the Methods section.*
☐	☒	A description of all covariates tested
☐	☒	A description of any assumptions or corrections, such as tests of normality and adjustment for multiple comparisons
☐	☒	A full description of the statistical parameters including central tendency (e.g. means) or other basic estimates (e.g. regression coefficient) AND variation (e.g. standard deviation) or associated estimates of uncertainty (e.g. confidence intervals)
☐	☒	For null hypothesis testing, the test statistic (e.g. F, t, r) with confidence intervals, effect sizes, degrees of freedom and P value noted<br>*Give P values as exact values whenever suitable.*
☒	☐	For Bayesian analysis, information on the choice of priors and Markov chain Monte Carlo settings
☐	☒	For hierarchical and complex designs, identification of the appropriate level for tests and full reporting of outcomes
☒	☐	Estimates of effect sizes (e.g. Cohen's d, Pearson's r), indicating how they were calculated

Our web collection on statistics for biologists contains articles on many of the points above.

Software and code

Policy information about availability of computer code

Data collection	No software was used.
Data analysis	All analyses were performed in R version 3.5.0 (R Foundation for Statistical Computing, Vienna, Austria, https://www.r-project.org/) and SAS 9.4 (SAS Institute Inc., Cary, NC, USA). The code used to generate these analyses are available on GitHub at https://github.com/JYoung2022FD/Sero-epi-data.git.

For manuscripts utilizing custom algorithms or software that are central to the research but not yet described in published literature, software must be made available to editors and reviewers. We strongly encourage code deposition in a community repository (e.g. GitHub). See the Nature Portfolio guidelines for submitting code & software for further information.

Data

Policy information about availability of data

All manuscripts must include a data availability statement. This statement should provide the following information, where applicable:

- Accession codes, unique identifiers, or web links for publicly available datasets
- A description of any restrictions on data availability
- For clinical datasets or third party data, please ensure that the statement adheres to our policy

The data that supporting the findings of this study are available on GitHub at https://github.com/JYoung2022FD/Sero-epi-data.git. All other data can be obtained from the corresponding author upon reasonable request due to privacy/ethical restrictions.

Human research participants

Policy information about studies involving human research participants and Sex and Gender in Research.

Reporting on sex and gender	The findings in our study do not apply to only one sex or gender. Sex and gender were not considered in the study design. Age and sex information were collected through face-to-face interview with caregivers. Of enrolled participants, 50.5% were boys in Children cohort and 54.0% were boys in Neonate cohorts. Sex-stratified analyses were conducted, while no gender-based analyses were performed in this study.
Population characteristics	Of the 2475 individuals in Children cohort, 50.3% (1246/2475) were boys. The baseline characteristics of boys were similar to that of girls, except birth weight (Supplementary Table 6).<br>A total of 1066 newborns were enrolled. Of these, 54.0% (576/1066) were boys.
Recruitment	For the 1-9 years of age group in Children cohort, the sample size for each study township was determined using the proportionate stratified sampling method. Within each township, simple random sampling was used for the selection of children aged 1-9 years.<br>Each study site generated a list of registered residents aged 1-9 years in the township who were eligible for enrollment. Considering the willingness to participate observed in the pilot study, 150% of the required number of enrollment will be randomly selected from the list. Potential participants were then approached and invited by well-trained project personnel and/or village doctors in order of random numbers. If selected children were unavailable or declined participation, the next eligible child was approached until sufficient children were successfully recruited in each age group. We approached 5,996 children and enrolled 4,188 (70%) children in total. We were unable to obtain the information of participants who were unavailable or declined participation, it was therefore impossible to directly evaluate the potential impact of participants enrollment. Whereas randomization procedure could have furthest reduced the risk of self-selection bias.<br>For Neonates cohort, newborns were eligible for inclusion if they were born after Sept 20, 2013, and their mothers had resided in the study sites in the preceding 3 months or longer. 3499 pregnant women were approached, 1054 (30%) of whom were enrolled and gave birth to 1066 paired neonates included in the study. We compared the characteristics of enrolled neonates with those from the same region who did not participate. Among neonates, sex and birthweight were similar.
Ethics oversight	Institutional review board approval was obtained from the Western Pacific Region Office of the World Health Organization (2013.10.CHN.2. ESR), China CDC (201224) and Fudan University (2019-05-0756), and written informed consent was obtained from all caregivers of participants.

Note that full information on the approval of the study protocol must also be provided in the manuscript.

Field-specific reporting

Please select the one below that is the best fit for your research. If you are not sure, read the appropriate sections before making your selection.

☒ Life sciences ☐ Behavioural & social sciences ☐ Ecological, evolutionary & environmental sciences

For a reference copy of the document with all sections, see nature.com/documents/nr-reporting-summary-flat.pdf

Life sciences study design

All studies must disclose on these points even when the disclosure is negative.

Sample size	A sample size of 553 participants for each group (including the 1-year, 2-year, 3-year, 4-year, 5-year, and 6-9-year age groups) would permit estimation of the incidence rate of EV-A71 infections as 10% with a 5% statistical significance level and 2.55% marginal error. Based on the pilot study, we assumed dropout rates of 21% for the 1-year and 2-year age groups and 15% for the other age groups. Accordingly, the adjusted sample sizes were 700 separately for the 1-year and 2-year age groups and 650 separately for the other age groups. We approached 5,996 children and enrolled 4,188 (70%) children in total. Of these, 50.5% were boys.<br>In addition, a longitudinal, paired mother–neonate cohort (Neonates cohort) was established from 23 September 2013 to 14 October 2015. A total of 1066 newborns were enrolled. Of these, 54.0% were boys.
Data exclusions	Completing the neutralising assays on neutralising antibodies against EV-A71 for all study participants is quite a resource-intensive task. For Children cohort, using multistage proportional stratified random sampling, we selected specimens from 50% of the enrolled participants aged 1-5 years for neutralising assays on neutralising antibodies against EV-A71. Stratification factors included 1) age (1 year, 2 years, 3 years, 4 years, and 5 years); 2) group (subgroup who were required to participate in semi-annual follow-up visits and others who participated in annual follow-up visits); and 3) number of follow-up visits. Supplementary Table 4 shows the distribution of participants aged 1-9 years. Considering the relatively small study population size in the 6- to 9-year-old age group, lab tests were conducted for all specimens.<br>In total, the specimens of 2475 participants were used for the lab test. The baseline characteristics of participants with and without lab tests in Children cohort were similar in terms of sex, ethnicity, and annual family income, but older children were overrepresented in the group that was tested.<br>Moreover, seven study participants in Neonates cohort were administered EV-A71 vaccines after 6 months of age during the study period; thus, their antibody titres after vaccination were excluded from this analysis.
Replication	Serum samples were inactivated at 56 °C for 30 min and then serially diluted 4-fold from 1:8 to 1:2048 with duplicate wells of each dilution.

Replication	Serum toxicity was determined by incubation of the lowest serum dilution with cells without virus. A cell control, positive antibody control and a virus back-titration without antibody were set up on each plate. All replications were successful.
Randomization	The randomization was used in the process of participants enrollment as well as samples selection for laboratory assays . The details have been described in above "Recruitment" and "Data exclusions" section. Briefly, for Children cohort, the sample size for each study township was determined using the proportionate stratified sampling method. Within each township, simple random sampling was used for the selection of children aged 1-9 years. Then using multistage proportional stratified random sampling, we selected specimens from 50% of the enrolled participants aged 1-5 years for neutralising assays on neutralising antibodies against EV-A71. While specimens from all children 6-9 years and neonates were tested for neutralising antibodies against EV-A71.
Blinding	There is only a unique number on the serum sampling tube, and the identification information of the participant was blinded to experimental operators.

Reporting for specific materials, systems and methods

We require information from authors about some types of materials, experimental systems and methods used in many studies. Here, indicate whether each material, system or method listed is relevant to your study. If you are not sure if a list item applies to your research, read the appropriate section before selecting a response.

Materials & experimental systems

n/a	Involved in the study
☐	☒ Antibodies
☒	☐ Eukaryotic cell lines
☒	☐ Palaeontology and archaeology
☒	☐ Animals and other organisms
☒	☐ Clinical data
☒	☐ Dual use research of concern

Methods

n/a	Involved in the study
☒	☐ ChIP-seq
☒	☐ Flow cytometry
☒	☐ MRI-based neuroimaging

Antibodies

Antibodies used	For quality control of laboratory assays on EV-A71 neutralising antibody, each testing plate included two positive antibody control wells. Positive antibody control (goat anti-rabbit antibody, working concentration: 1:1024) were provided by the company “Sinovac Biotech”.
Validation	Sinovac Biotech, one of the manufacturers for EV-A71 vaccines, provided the polyclonal antibody which was generated by immunizing rabbits with the purified EV-A71 intact virion. We used two EV-A71 strains (C4a and C4b) to validated the antibody, it shows good neutralization reaction.
